# Prenatal screening of cytogenetic anomalies – a Western Indian experience

**DOI:** 10.1186/s12884-015-0519-y

**Published:** 2015-04-12

**Authors:** Frenny Sheth, Mizanur Rahman, Thomas Liehr, Manisha Desai, Bhumika Patel, Chirag Modi, Sunil Trivedi, Jayesh Sheth

**Affiliations:** FRIGE’s Institute of Human Genetics, FRIGE House, Satellite, Jodhpur Gam Road, 380015 Ahmedabad, India; Institute of Human Genetics, Jena University Hospital, Kollegiengasse 10, D-07743 Jena, Germany

**Keywords:** Karyotyping, GTG-banding, Fluorescence in situ hybridization (FISH), Array-comparative genomic hybridization (aCGH), Cell free DNA in maternal circulation (cfDNA), Chromosomal abnormalities, Prenatal samples

## Abstract

**Background:**

Children born with congenital anomalies present a very high rate of perinatal death and neonatal mortality. Cytogenetic analysis is a convincing investigation along with clinical suspicion and biochemical screening tests. The current study was designed to characterize the prevalence and types of chromosomal abnormalities in high risk prenatal samples using different cytogenetic techniques.

**Methods:**

This study was conducted on a total of 1,728 prenatal samples (1,324 amniotic fluids, 366 chorionic villi and 38 cord blood samples) from 1994 to 2014 at Institute of Human Genetics, Ahmedabad, India. Conventional karyotyping was conducted with GTG-banding. Molecular approaches were used (fluorescence in situ hybridization = FISH and/ or array-comparative genomic hybridization = aCGH) when indicated to detect karyotypic abnormalities.

**Results:**

Abnormal karyotypes were detected in 125/1,728 (7.2%) cases. Trisomy 21 was the most common abnormality detected in 46 (2.7%) followed by trisomy 18 in 11 (0.6%) and trisomy 13 in 2 (0.1%) samples. Besides, structural abnormalities such as reciprocal and Robertsonian translocation were detected in 20 [1.2%] cases. Turner syndrome was diagnosed in seven (0.4%) cases; in six (0.34%) cases there was an inversion in the Y-chromosome. Heteromorphic variants were diagnosed in 22 (1.3%) cases. Finally, small supernumerary marker chromosomes (sSMC) were found in six (0.34%) cases.

**Conclusion:**

Conventional GTG-banding along with molecular cytogenetic techniques is useful in detecting genomic alterations and rearrangements. Comprehensive characterization of chromosomal rearrangements like sSMC has the potential to save potentially healthy fetuses from being terminated.

## Background

Naturally, the most expressed desire of a couple is to have a physically and mentally healthy baby after being pregnant. Obviously, any screening program that aims to provide contributions for such an assurance will cause anxiety while waiting for the test results. False positive screening tests in sonography and/or maternal serum screening and lack of therapeutic options for chromosomal abnormalities will cause additional problems. It is therefore important to select appropriate screening tests and diagnostic procedures that are precise, accurate, safe and can be performed during early pregnancy to achieve the most meaningful decision considering the potential outcome of pregnancy. Approximately 2.5% of infants are born with congenital anomalies; accounted for 8-15% of perinatal deaths and 13-16% of the neonatal mortality in India. Chromosomal anomalies are detected in 6% of them [1,2]. The most common chromosomal abnormalities are numerical ones of the sex chromosome and for the autosomes (trisomies 21, 18 and 13). While point mutation rate is slightly higher in sperms of older fathers; the risk for trisomies is significantly increased with advanced maternal age. Average maternal age at child bearing has increased throughout the world; also the prenatal diagnostic facilities have improved tremendously which ease to make a decision on the fate of pregnancy [3].

Among the prenatal screening tests, ultrasonography is the simplest available non-invasive method for detection of abnormal embryogenesis (increased nuchal translucency, double bubble sign, duodenal atresia, spina bifida etc.). Double marker [pregnancy-associated plasma protein A (PAPP-A) and free ß-HCG (human chorionic gonadotropin)], triple marker [α-feto protein (AFP), free βHCG, unconjugated estriol (μE)] and/or quadruple marker screens [4] on maternal serum or in combination with ultrasonography can be applied to identify fetuses at risk of autosomal aneuploidies, particularly trisomies 21, 18, 13. They are still only ‘risk-determination-tests’ as a definite chromosomal diagnosis can be made only from the fetal cells. Fetal cells can be safely obtained from amniotic fluid (AF), chorionic villus sampling (CVS) or cordocentesis (CB) and can be used for precise and accurate prenatal diagnosis [5].

Second trimester studies of AF are performed at about 16 weeks’ of gestation without significant technical difficulties. However, alternative options include CVS at 11-13 weeks’ and early amniocentesis [6]. Very recently, fetal cells can be studied from maternal blood, more precisely from fetal DNA in maternal circulation (cfDNA) with a limitation of detecting most common aneuploidy like trisomy 21, 18, 13 only [7]. Similarly, alteration at the submicroscopic level across the genome can be studied using array Comparative Genomic Hybridization (aCGH) from fetal DNA [8].

The aim of the present study was to assess the prevalence and types of chromosomal abnormalities in cases where ultrasonography and biochemical markers were positive, advance maternal age or history of genetic disorders in the family were reasons for fetal cell acquisition. The samples were collected over a period of 20 years in Gujarat, Western India and primarily studied by conventional banding cytogenetics. Moreover, the advantages and limitation of various molecular cytogenetic techniques in detecting genomic rearrangements are discussed.

## Methods

This retrospective observational cross-sectional study was carried out at Institute of Human Genetics for the period between 1994 and 2014. A total of 1,728 prenatal samples (cases) were included in the study [amniotic fluid (n = 1324), chorionic villus (n = 366) and cord blood (n = 38)]. The patients were adequately counseled regarding prenatal diagnosis after obtaining family and gestation history. Signed informed consent was obtained as per Helsinki declaration for utilization of the patient information and data for academic purposes including research publications. Approval to carry out the study was acquired from FRIGE institutional ethics committee. Amongst the 1,728 cases investigated, 892 prenatal cases were positive in biochemical screen, 227 were positive for soft markers through ultrasonography, 559 cases presented with advanced maternal age, and 50 cases provided a history of previous children with genetic disorders.

After receiving the samples, they were processed as per the standard protocol of GTG-banding to determine the karyotype. Fluorescence in situ hybridization (FISH) and/ or array-comparative genomic hybridization (aCGH) studies were carried out following standard approaches as and when indicated.

## Results

**Figure 1:**
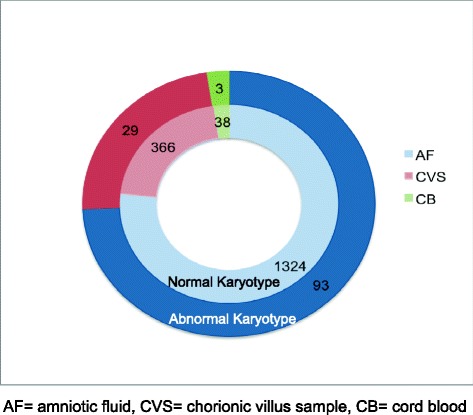
Number of cases (both normal and abnormal) in different prenatal samples.

The mean age of all participating women was 31.6 years (range: 19 to 53 years). Chromosomal abnormalities were detected in 125 (7.2%) cases. Most likely disease causing chromosomal abnormalities was found in 103 (6%) cases and heteromorphic variants were detected in 22 (1.3%) cases. The distribution of chromosomal abnormalities detected in different samples is shown in Figure 1. Chromosomal alterations in respect to various risk factors are depicted in Table 1. Among 125 cases of chromosomal anomalies, 50 (40%) cases had positive biochemical marker screen test, 17 (13.6%) cases were studied due to advanced maternal age, 32 (25.6%) cases were associated with positive soft markers and 26 (20.8%) cases had previous history of children with genetic disorders.

**Table 1:** **Distribution of chromosomal abnormalities with respect to risk factors (n = 125)**

**Sample**	**Numerical**	**Structural anomalies**						
		**Robertsonian translocation**	**Reciprocal translocation**	**sSMC**	**Isochromosome**	**Deletions**	**Inversion Y**	**Variant**
**amniotic fluid (n = 93)**								
AMA	5	-	2	2	-	1	2	1
TMS positive	23	3	2	-	-	1	2	4
soft marker positive	17	1	3	-	-	-	-	6
H/O of genetic disease	4	2	2	4	-	-	1	5
**chorion (n = 29)**								
AMA	1	-	1	-	1	-	1	-
TMS positive	10	-	1	-	-	-	-	2
soft marker positive	4	-	-	-	-	-	-	-
H/O of genetic disorder	2	2	1	-	-	-	-	3
**cord blood (n = 3)**								
AMA	-	-	-	-	-	-	-	-
TMS positive	1	-	-	-	-	-	-	1
soft marker positive	1	-	-	-	-		-	-
H/O of genetic disease	-	-	-	-	-	-	-	-

AMA = advanced maternal age; H/O = history of; TMS = triple marker study.

Table 2 shows the summary of the chromosomal aberrations detected. The most common numerical alteration diagnosed was trisomy 21 (46/1728; 2.7%), followed by trisomy 18 (11/1728; 0.6%), monosomy X (7/1728; 0.4%) and trisomy 13 (2/1728; 0.1%); triploidy and tetraploidy were detected in one case, each. Finally structural anomalies were detected in 2.02% (35/1728) and heteromorphic variants in 1.3% (22/1728) of the cases (Table 2).

**Table 2:** **Summary of chromosomal aberrations (n = 125)**

	**Chromosomal abnormalities**	**Number**
numerical anomalies* (n = 68)	47,XN,+13	2
	47,XN,+18	11
	47,XN,+21	41
	45,XN,-21	1
	46,XN/47,XN,+21	1
	45,X	7
	45,X/46XN	1
	46,XN/47,XN,+2	2
	69,XNN	1
	92,XNNN	1
**structural anomalies (n = 57)**		
reciprocal translocation (n = 12)	46,XN,t(2;20)(q23;q13.1)	1^@^
	46,XN,t(2;21;4)(p12;q21;q34)	1
	46,XN**,**t(4;16)(p14;q13)	1
	46,XN,t(7;21)(p32;q22)	1
	46,XN,t(9;10)(q13;q11.2)	1
	46,XN,t(11;22)(q13.2;q11.2)	1
	46,XN,t(12;18)(p11.2;q11.2)	1
	46,XN,t(16;18)(p13;q11)	1
	46,XN,t(18;19)(p11.2;p13)	1
	46,XN,t(X;7)(p22.2;p21)	1
	46,XN,add(2)t(2;10)(p23;q22)	1
	46,XN,der(9)t(9;?)(q13;?)	1***
Robertsonian translocation (n = 8)	45,XN,rob(13;14)(q10;q10)	2^#^
	45,XN,rob(21;22)(q10;q10)	1
	45,XN,rob(14;15)(q10;q10)	1
	46,XN,rob(13;21),+21	1
	46,XN,rob(14;21),+21	2
	46,XN,rob(21;21)(q10;q10),+21	1
deletion (n = 2)	46,XN,der(X),del(X)p11	1
	46,XN,del(16)(q13;q22)	1
sSMC (n = 6)	47,XN,+mar.ish inv dup(Y)(pter- > Yp11.2::Yp11.2- > pter)	1
	48,XN,+inv dup(13 or 21)(q10),+del(13 or 21)(q10)	1
	47,XN,+mar	2**
	46,XN/47,XN,+mar	2**
isochromosome (n = 1)	46,XN,i(X)(q10)	1
inversion (n = 6)	46,X,inv(Y)	6
variant (n = 22)	Satellite and heterochromatic polymorphism	22

* Four cases initially showed chromosomal aberrations and later on found as normal in second sample, hence they are not included the study as abnormal.

** Parents opted for termination and no further characterization was done.

*** Patient declined for further analysis.

^#^ Detail history in case 8.

^@^ Except 46,XN,t(2;20)(q23;q13.1) all the translocations were derived from one of the parents.

Mosaics were initially detected in four cases but further examination of second samples later during pregnancies or at birth revealed normal karyotypes, hence these cases were considered as normal. Six (0.34%) carriers of sSMC were detected and in four cases parents declined further analysis and opted for immediate termination of pregnancy. Hence, precise characterization of sSMC was carried out only in two cases (Table 2).

Selected cases are described below. They demonstrate importance of karyotyping and molecular cytogenetics followed by genetic counseling to provide a good care for pregnant women.

### Case 1

The triple marker screen in a 30 year-old female was 1:3,000 for trisomy 21 with an age related risk for aneuploidy of 1:700. Considering individual screening markers, there was the following observation; β-HCG = 0.7 MoM; μE = 0.7 MoM; AFP = 1.0 MoM. The amniotic fluid study was then conducted in view of a possibility for cytogenetic anomaly [4]. Interphase-FISH (I-FISH) on uncultivated amniocytes excluded trisomies for chromosomes 13, 18 and 21. Chromosomes derived from cultured amniotic cells revealed a karyotype 46,X,i(X)(q10). This demonstrates that structural abnormality can also be present in triple marker screen and I-FISH negative cases. Thus interpretation of individual biochemical parameters is important and one should not depend only on software based risk calculations.

### Case 2

Amniotic fluid cells from a 38 year old female were analyzed as triple marker screen showed an increased risk for trisomy 21 (1:200) with AFP = 2.3 MoM, β-HCG = 2.0 MoM and μE = 0.8 MoM. The age related risk for aneuploidy was 1:150. I-FISH in uncultivated amniocytes excluded trisomies for chromosomes 13 and 21. Chromosome analysis from cultivated amniotic cells showed a karyotype 46,X,del(X)(p11). This demonstrates that structural abnormality can be present in triple marker screen positive and I-FISH negative cases. It indicates that I-FISH should be used only to relieve patient’s anxiety and not as a single confirmatory test; a confirmation is to be sought by comprehensive chromosomal studies from cultivated cells.

### Cases 3 to 5

Three pregnant women with an age range of 22 to 30 years were referred for karyotype studies from AF due to fetal anomalies detected in ultrasonography during late second or mid third trimester. Different soft markers like double bubble sign and hyperechoic lesions in both the ventricles or in one each, while duodenal atresia and limb abnormalities was detected only in one case. Triple marker screening test was not carried out in any of these cases. Chromosomal analysis from AF in these three cases revealed trisomy 21, 13 or 18 respectively which could have been detected quite early if double or triple marker screening test would have been carried out.

### Case 6

Prenatal study was advised to an elderly couple during primi gravida. Kayotype analysis was carried out from AF that showed the presence of two de novo sSMCs; karyotype 48,XN,+mar1,+mar2[100%]. Triple marker study carried out at 16 weeks of gestation did not show any enhanced risk for trisomy 13, 18 and 21. Fetal anomaly scan carried out at 16 and 24 weeks were normal. After application of FISH probes developed for sSMC-characterization described elsewhere [9]; the sSMCs were characterized as inv dup(13 or 21)(q10) and del(13 or 21)(q10). As the sSMCs were exclusively made up of heterochromatic material they were unlikely to be associated with any clinical abnormalities. Most likely, the sSMC are derived from each other. Advanced maternal age was most often associated with sSMC derived from #13/21 and in majority of cases, no dysmorphism was detected after birth. The pregnancy in the patient therefore was continued and resulted in the delivery of a normal healthy child.

### Case 7

**Figure 2:**
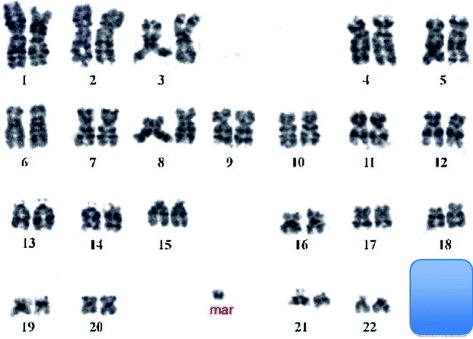
Karyotype of case 7 showed presence of sSMC in addition to normal 46 chromosomes.

Prenatal diagnosis was offered during third gravida to a young couple. They had a previous child with free trisomy for chromosome 21 [Down syndrome] and her second pregnancy ended in a first trimester miscarriage. Fetal karyotype obtained from AF revealed presence of a ‘*de novo*’ sSMC in all cells with normal sex chromosomes karyotype: 47,XN,+mar[100%] as parental chromosomal study was normal [Figure 2]. Detailed molecular cytogenetic characterization was carried out with the final karyotype 47,XN,+mar.ish inv dup(Y)(pter- > Yp11.2::Yp11.2- > pter)(SRY++)(subtelX/Y++). Thus, a neocentric sSMC formation was observed. This case showed repeated aneuploidy and the sSMC would have been missed if only I-FISH test was carried out.

### Case 8

A young non-consanguineous couple had two first trimester fetal losses and two children with congenital anomalies and mental retardation of varying degrees. Chromosome analysis of both the sibs (7 yrs and 5 yrs) was normal at 550 band resolution. During 5^th^ gravidity, a CVS sample was processed for cytogenetic study. It revealed a Robersonian translocation involving chromosomes 13 and 14 inherited from the phenotypically normal father. Array Comparative Genomic Hybridization (aCGH) analysis showed 151-283 kb deletion inside *LINGO2* (*LRRN6C*) gene on 9p21.1 in the affected sibs, in CVS and the father. Since the father was phenotypically normal, this deletion is most likely a CNV (copy number variant) without phenotypic effects. Even though all the analyses were normal, parents opted for termination of pregnancy due to array report.

## Discussion

Data derived from population-based congenital anomaly registries show that many fetal miscarriages or intrauterine death are due to chromosomal abnormalities in the first trimester of pregnancy; being detected as a consequence of advent of prenatal screening [10]. In the present study, chromosomal anomalies were detected in 125 (7.2%) prenatal samples. This high number of chromosomal abnormalities in our study is likely to be due to a selection bias of investigating only highly suspected referral cases with possible chromosomal abnormalities. This rate therefore was higher than that found in consecutive unselected newborn studies (e.g. 0.262 per 100 cases) [11,12]. The 7.2% abnormal cases that were classified as rare microscopically visible chromosomal abnormalities (such as deletions, trisomies, unbalanced translocation, markers, mosaicism and apparently balanced rearrangements) are much lower than the study reported by Baena et al. [13].

In all recognized conceptions, triploidy is estimated to follow in 1–2% [14] with a miscarriage rate of 33% before 15 weeks of gestation. In this study one tetraploidy and tripoloidy each was detected during second trimester.

sSMCs were found in six (0.4%) cases in the current study. Liehr and Wiese reviewed 132 studies on sSMC with an average prevalence rate of 7.5/10,000 births [15]. The prevalence rate in the present study was higher which most probably was due to selection of only referral cases as well as smaller sample size. Both cases were comprehensively characterized further by molecular cytogenetics. If aCGH would not have been used, case 7 would have been missed as only presence of heterochromatin on the sSMCs.

Chromosomal deletions in prenatal samples are rare [16,17] as well. Congenital anomaly register data showed the prevalence of microscopically visible deletions in the range of 0.3 to 2/10,000 births [18-20]. In the present study, only two such cases were found. Overall, it is difficult to find out the prevalence rates in a country like India where the rate of illiteracy is high with less priority to health issues.

In summary, 3.4% cases were detected with trisomies of chromosomes 13, 18 or 21 (59/1,728) while in 3.7% cases, other chromosomal rearrangements were present. As fetal loss is the most common fear among women while undergoing invasive prenatal procedure, recent detection of cfDNA is a promising technique; offering the possibility to detect fetal numerical chromosomal aberrations in a non-invasive way [21]. In invasive prenatal diagnosis, another relatively new investigative tool with genome wide coverage (aCGH) and high resolution at molecular level revolutionized cytogenetics [22]. In the present study, 20 translocations (12 reciprocal and 8 Robertsonian ones) were detected. Apparently balanced translocation was observed in all these cases and no dysmorphism was detected after birth except in three cases (trisomy 21). These genomic rearrangements [i.e. der (13;14)] would have been missed by aCGH or noninvasive technique of fetal blood in maternal circulation (cfDNA) as shown in case 8. Nonetheless, aCGH increases the detection rate of genomic imbalances by 2.9% over conventional karyotyping [23]. However, chromosomal microarray in prenatal testing cannot substitute conventional karyotyping as a tool for general screening in all pregnancies. ACOG committee [8] however, recommends chromosomal microarray in patients undergoing invasive prenatal diagnosis and this test replaces the need for fetal karyotype when structural anomalies are detected under ultrasonography examination. Secondly, a genetic mutations identified by chromosome microarray study is not confined to advanced maternal age. In India, the availability of chromosomal microarray is very limited and expensive. It is accessible only to a very small number of clinicians and centers offering this test. Fetal karyotype from the samples obtained by invasive methods therefore still continues to be a widely accepted modality in such cases. The need for pre-test and post-test genetic counseling from qualified personnel has to be strictly followed [8]. However, looking to our data, nearly 50% cases would have been missed using this technology only, which indicates that karyotype studies from CVS or AF are the gold standard; patients should therefore be counseled and informed about the advantage and limitation of aCGH and non-invasive technologies. By detecting abnormal karyotypes during prenatal period, it is possible to reduce the rate of chromosomal abnormalities among the populations of a country; thereby reducing social as well as family trauma and financial burden due to disabled children. It must still be an individual decision of the pregnant woman and her partner if they decide for or against a child with a known chromosomal abnormality. The society also has to accept and support decisions for disabled children due to religious or other reasons as abortions are also known to cause trauma to the affected woman.

## Conclusion

In the era of aCGH and next generation sequencing, conventional karyotyping maintains its role as a diagnostic tool in detecting gross chromosomal alterations and rearrangements. However, in the cases of sSMCs, molecular techniques further complement in characterizing chromosomal rearrangements that can save potentially healthy fetuses from being terminated. Additionally, cryptic imbalances can be detected with aCGH when structural anomalies are detected during an antenatal ultrasonography examination.
